# Mucocutaneous presentation of Kaposi sarcoma of rapid growth in a young male with HIV infection

**DOI:** 10.11604/pamj.2020.36.194.22965

**Published:** 2020-07-20

**Authors:** Luis Alberto Pérez-Arredondo, Jorge Rafael Violante-Cumpa

**Affiliations:** 1 Universidad Autonoma de Nuevo Leon, Facultad de Medicina y Hospital Universitario “Dr. Jose Eleuterio Gonzalez”, Departamento de Medicina Interna, Monterrey, Nuevo León, México

**Keywords:** Kaposi sarcoma, mouth neoplasms, HIV infection

## Image in medicine

A 26-year-old male with a medical history of risk sexual behavior, is brought to the Emergency Room (ER) with complaints of dyspnea associated with an oral cavity mass. At the interrogation, he referred that the symptomatology started 2 months ago with a lesion of approximately 1cm, originated from the upper gum. Patient also reported dysphonia, and dysphagia due to the expansion of the mass, and a weight loss of 10 kilograms. At physical examination a purple-black colored mass was identified coming from the oral cavity (A), including the upper gums with extension into the palate, reaching the uvula (B), the mass had exophytic margins, no tenderness at palpation with a fetid smell, multiple maculopapular violaceous lesions of approximated 1cm located in the neck, thorax and upper extremities. At palpation, bilateral cervical and inguinal adenopathies were detected, blood pressure within range, tachycardic, tachypneic but with oxygen saturation of 94% at room-air and a body temperature of 36.8°C. The laboratory tests reported anemia and thrombocytopenia, hyponatremia and an antigen-antibody test positive to HIV. A skin biopsy from skin violaceous lesions in forehead reported a positive result for Human Herpes Virus 8, within the next 48 hours the patient started with fever and hypoxemia (So_2_84%), antibiotic therapy was started with levofloxacin under suspicion of bacterial pneumonia, within the next day the patient presented hemoptysis; thus, a bronchoscopy was ordered. During the bronchoscopy the patient exhibited hypotension, despite intravascular fluid resuscitation; the patient entered cardiac arrest with no response to cardiopulmonary resuscitation.

**Figure 1:**
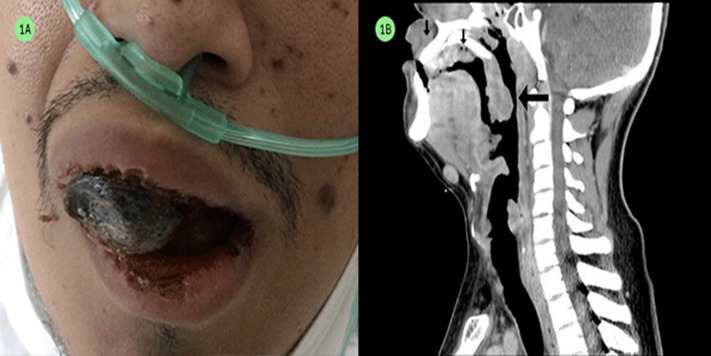
A) mucocutaneous Kaposi sarcoma; B) computed tomography in sagittal plane of oral cavity and the Kaposi sarcoma extension (arrows)

